# A Lightweight Web of Things Open Platform to Facilitate Context Data Management and Personalized Healthcare Services Creation

**DOI:** 10.3390/ijerph110504676

**Published:** 2014-04-29

**Authors:** Iván Corredor, Eduardo Metola, Ana M. Bernardos, Paula Tarrío, José R. Casar

**Affiliations:** Data Processing and Simulation Group, School of Telecommunication Engineering, Universidad Politécnica de Madrid, Avda. Complutense 30, 28040 Madrid, Spain; E-Mails: eduardo.metola@grpss.ssr.upm.es (E.M.); abernardos@grpss.ssr.upm.es (A.M.B.); paula@grpss.ssr.upm.es (P.T.); jramon@grpss.ssr.upm.es (J.R.C.)

**Keywords:** open platform, web of things, user context monitoring, resource-oriented architecture, sensor fusion

## Abstract

In the last few years, many health monitoring systems have been designed to fullfil the needs of a large range of scenarios. Although many of those systems provide good ad hoc solutions, most of them lack of mechanisms that allow them to be easily reused. This paper is focused on describing an open platform, the micro Web of Things Open Platform (*µ*WoTOP), which has been conceived to improve the connectivity and reusability of context data to deliver different kinds of health, wellness and ambient home care services. *µ*WoTOP is based on a resource-oriented architecture which may be embedded in mobile and resource constrained devices enabling access to biometric, ambient or activity sensors and actuator resources through uniform interfaces defined according to a RESTful fashion. Additionally, *µ*WoTOP manages two communication modes which allow delivering user context information according to different methods, depending on the requirements of the consumer application. It also generates alert messages based on standards related to health care and risk management, such as the Common Alerting Protocol, in order to make its outputs compatible with existing systems.

## 1. Introduction

Nowadays, an increasing number of personal health sensors are commercially available: from innovative activity quantifiers, sleep monitors or mood trackers, to biometric solutions, such as blood pressure or under-skin glucose monitors or wearable dopplers. In this nascent market, a lot of technology providers coexist; they implement their business strategy and customer attraction through different models, in many cases, are built around a offering of the hardware and a closed application to facilitate data visualization. In the best of cases, a proprietary non-standard API enables third parties to develop new applications. Most of these providers have developed their value proposals around cloud services, thus sensor data are managed through the Internet, and exposed through different type of services to consumers. We could say that this is a practical example of the implementation paradigm of Internet of Things. According to it, everyday objects may connect to an Internet-like network [1] without a common standardized interface to access to services offered by them.

Obviously, this scenario presents a very fragmented approach that makes difficult handling data from different sources without dedicating a non-negligible effort to integration tasks. It happens that advanced context-aware applications (for example, those in the area of health, such as behaviour monitors, fall detectors, automatic diary builders, *etc.*) may need to coordinate and synchronize information retrieval from different sensors, which can be stand-alone but also smartphone-embedded ones, or may be deployed on infrastructure settings (e.g., hybrid wireless sensor networks). Additionally, many services or applications may require to control the the environment through actuators.

In these circumstances, how to effectively handle a variable amount of heterogeneous sensor and actuator elements is still an open challenge. In particular, research is now dealing with different architectures and structures to enable the *Web of Things*, an evolution of the IoT paradigm, which aims at interconnecting devices into the Internet as Web resources, using traditional Web standards (such as HTTP, REST, URI, *etc.*) to facilitate accessing the objects’ capabilities [2].

In this paper we delve in how to facilitate the ubiquity, reusability and interoperability of Web of Things platforms, in particular by analysing the requirements that those services for personalized health, wellness and ambient home care pose. These services differ in their sensing infrastructure, real time needs, querying modes, *etc.* To address these issues, we propose the “micro Web of Things Open Platfor” (*µ*WoTOP), which provides an architecture that facilitates both connecting external sensors and developing consumer application by using REST interfaces and the publisher-subscriber paradigm. *µ*WoTOP facilitates several modes for information consumption and makes easier the integration of new sensing devices. In order to enable interoperability, the proposed system is prepared to host specific parsers that will wrap messages in different well-known standard formats, such as the Common Alerting Protocol (CAP) for alerts or HL7 or SNOMED for clinical interoperability. *µ*WoTOP has been implemented for trial and validation to run on Android platforms in order to guarantee its ubiquity, offering enough good performance for the type of health-oriented applications that are considered in this paper.

The remaining of this paper is structured as follows. Section 2 reviews the state of the art in Web of Things platforms. Section 3 analyses in detail which type of capabilities are expected from a platform that aims at being light, ubiquitous, interoperable and reusable. This analysis is done from an application perspective. Section 4 states the design principles of *µ*WoTOP and describes its architecture, together with the workflow that a developer should have to follow in order to design an application on top of it. Section 5 motivates a case of use for *µ*WoTOP, in which it is detailed the design of a system to promote independent living of an elderly at his place or in a residence. Section 6 analyses the performance of *µ*WoTOP for this case of use, in terms of real time and reliability behaviour. Conclusions and further work are in Section 7.

## 2. Related Work

Currently, many personal devices (e.g., smart phones, smart watches or sport monitoring bracelets) are equipped with inertial sensors (e.g., accelerometers, gyroscopes), biometric sensors, localization sensors, *etc.* These devices have become pervasive alerting platforms for many different scenarios, such as health monitoring, patient rehabilitation and sports training control. In particular, their use to detect and alert about health emergency situations is attracting the interest of the commercial scene [3]. For example, special attention is being put on notification systems that dispatch messages in real time [4], in particular, fall detection [5] and early risk detection [6]. Many of these systems are built on an ecosystem of heterogeneous sensors made by different manufacturers, so that they are set up according to their own hardware and software components. This situation motivates the necessity of managing healthcare systems through a global, open platform.

There is no single standard aimed at implementing interoperable health services. As with technologies already integrated in everyday life (email, mobile or Web) interoperability among health systems do not accomplish a *one-size-fits-all* solution. On the opposite, it is quite usual that different providers use their own technologies that are incompatible among them.

Several organizations and consortia are focused on reaching unified guidelines to develop and harmonize standards and specifications which could motivate the design of open platforms for healthcare. For instance, the Office of the National Coordinator for Health Information Technology (ONC) [7] of the USA has initiatives to specify a guidance to solve interoperability issues. Those under-way initiatives are organized in the following working groups: *meaning*, *structure*, *transport*, *security* and *services*.

The Healthcare Information and Management Systems Society (HIMSS) [8] defined the interoperability in healthcare as “the ability of different information technology systems and software applications to communicate, exchange data, and use the information that has been exchange” [9]. HIMSS organizes health information interoperability into three levels, from greatest to least interoperability: *foundational*, *structural* and *semantic*.

Continua Health Alliance, that forms the Personal Connected Health Alliance with HIMSS, is also working on designing guidelines [10] to facilitate the deployment of personal connected health devices in order to promote the incorporation of health and wellness into the everyday lives of users. Continua’s guidelines establish standard-based technologies to connect health systems: sensors, tablets, gateways and smartphones as well as cloud services. Such device ecosystem is wirelessly connected by Bluetooth Low Energy and ZigBee wireless protocols. Recently, IEEE Standards Association (IEEE-SA) and Continua Alliance have signed an agreement to expand the use of the ISO/IEEE 11,073 personal health device (PHD) family of standards in order to reduce the gap across the wide variety of personal health devices.

ISO/IEEE 11,073 (PHD) standards address a wide range of technical issues. Among these issues, it has to be highlighted their nomenclature and terminology, compatible with SNOMED, that is optimized for managing vital sign data based on an object-oriented data model. On the other hand, ISO/IEEE 11073 specifies and inter-networking standard in order to facilitate messaging between systems based on different data representation standards as HL7. The latter feature can become a big step towards a whole interoperable solution since, currently, HL7 is the most popular standard for electronic health information. Specifically, HL7 v2 was a well adopted standard in health care institutions on a worldwide scale for more than a decade, building the pillars of open platforms for messaging [11] or EHR systems [12].

Other initiatives are focused on providing unstructured information. A popular option to carry out such a data schema is Linked Data that uses Web standard technologies (HTTP, RDF, URIs) instead of proprietary models. Currently, these type of initiatives are being promoted through projects such as the Open Source Electronic Health Record Agent (OSEHRA) [13] or Fast Healthcare Interoperability Resources (FHIR). Specifically, the latter is an HL7’s initiative that is working on a simplified version of HL7 v3. FHIR has included the specification of RESTful interfaces to manage health information resources. Thus, every medical concept is defined by a URI and the data is formatted in XML or JSON documents.

It is essential that all the standardization and harmonization processes, described previously, are orchestrated and offered through a well-specified service policy. These service premises should allow building health information systems through transport and structure agnostic technologies. In this sense, The Web of Things paradigm [2] has been proposed as a solution to fulfill the interoperability of health systems needs at service level as defined by ONC. The key feature of the WoT is to wrap embedded devices using Web interfaces in order to remove communication barriers between virtual and real worlds. WoT-based solutions are usually divided into two different categories [2]: (i) directly integrated into the Web; and (ii) indirectly integrated into the Web.

On the one hand, integrating smart things directly into the Web requires that all the smart things have IP connectivity as well as an embedded Web server is implemented in every smart thing. We have already explored such approach in our previous work [14], which proposed a Knowledge-Aware and Service-Oriented (KASO) middleware that provides access points to expose sensors and actuators as REST resources. Likewise, Guinard *et al.* [15] proposed a RESTful prototype consisting in running an embedded Web server on Sun SPOT nodes. In this way, the node’s sensors can be accessed by invoking a GET HTTP method directly over a URI offered by a Sun SPOT. In the same line, Vazquez *et al.* [16] suggested the deployment of a tiny HTTP client on an embedded platform. In general, the main advantage of this type of approach is the low latency that is introduced when getting a result from resources provided by embedded web servers. On the contrary, the weakest point is the lack of scalability, which is related to the constraints of the embedded devices in terms of energy, CPU or memory. This makes it difficult to offer services guaranteeing a minimum level of Quality of Service (e.g., service availability or reliability).

On the other hand, the indirect integration of smart things into the Web is done through an intermediate proxy or smart gateway that manages isolated networks of embedded devices which are based on privative technologies. The function of these smart gateways is to connect embedded devices or smart things with the Web. This is achieved by implementing at least two protocol stacks: a full TCP/IP protocol stack to communicate with Internet-connected entities, and a proprietary protocol stack (e.g., Zigbee or Bluetooth) to communicate with networks of embedded devices. Smart gateways typically use uniform interfaces to expose the functionalities of the different smart things. An example of this type of system is described in Trifa *et al.* [17]. This work propose a smart gateway that integrated embedded devices into the Web through a system based on drivers. Another example was proposed by the SmartThing [18] project, which has recently launched a SmartThings Hub, a smart gateway exposing embedded and customizable devices to be mapped over RESTful interfaces.

Another common solution is to offer WoT platforms according to cloud computing premises applying the Platform as a Service (PaaS) paradigm. The PaaS model enables service consumers to easily develop and deploy value-added services by using workbench tools provided by a vendor. In this way, few resources are spent for management and maintenance of large infrastructures of hardware and software. Most PaaS-based solutions have some common features such as the provisioning of a deployment environment, monitoring dashboard, and communication mechanisms to exchange information between the platform and clients. Computing resources transferred to client accounts usually depends on the scalability and Quality of Service required by the applications using the platforms. Xively [19], EVRYTHNG [20], Paraimpu [21] or ThingSpeak [22] are standard examples of PaaS-based services. These platforms provide RESTful services through which information can be managed only by accessing URIs that identify each data stream, usually called feeds or channels. Services provided by these platforms are specially focused on those systems needing high scalability levels. However, they strongly depend on cloud services and, thus, on the Internet infrastructure. Therefore, the main weaknesses of this type of solutions are the lack of security in information transactions, and unpredictable latencies in the notification of events to the clients.

To overcome the above mentioned drawbacks, we propose the micro-WoT Open Platform (*µ*WoTOP), a lightweight WoT platform, which offer a set of functionalities and tools that facilitates to build enriched ecosystems of networked smart things using web technologies as interoperability mechanisms. In particular, our platform has been designed to fulfill the requirements of different applications for personal care, wellness and ambient assisted living, as it is explained in the next Section.

## 3. Motivation for an WoT-Based Open Platform

### 3.1. An Overview of Service Needs

An open WoT-based platform designed to deal with health-related context data must be able to fulfill a number of requirements, guaranteeing the offering of reliable data among diverse applications, each of them characterized by different needs (e.g., in terms of performance, scalability or information formats). In order to determine these requirements, we first analyse the features of some services that could be deployed on top of the platform:

(1) **A fall/faint detector for elderlies living in a small retirement home,** which dispatch alerts to caregivers if an elder falls or faints. In this scenario, a single caregiver may have to supervise a number of elders (e.g., up to 100). Additionally, elders may be comfortable to know that their family or flatmates are notified in case that a fall happens. The sensing technology to determine a fall may vary from an inertial sensor placed in the elder’s chestbelt to a wristwatch. Additional sensors (e.g., skin temperature and humidity) in intelligent cloths can provide complementary information that may help to discriminate the cause of the fall. These sensors may have computing capabilities and generate processed alerts or raw sensing data, to be fused within specific external components.
(2) **A routines analyzer that alerts about risky situations for a person living alone.** This background system is able to detect abnormalities in the behavior of a person (e.g., eating and movement habits). Its service objective is to provide family members or caregivers with deviation alerts of the user’s normal habits. Routine analysis may be performed on location, activity (movement) and use of objects data, which are the input data for the analyzer. On a deviation alert (e.g., showing depression, malnutrition or sendentariness), the family member may actively check the situation to confirm that everything is going right.
(3) **Daily medical check-up system at home.** An elder at home may register his daily health parameters (collected through personal wearable or infrastructure sensors) and provide general information about his health status (mood, energy feelings, *etc.*). These data will be sent to an external repository, in a medical standard format, for the elder’s doctor to retrieve and evaluate them in a posterior telemedicine session. No time-constraints are to be handled in this service scenario, as the quality of the collected data may be checked and the user may collaborate in case that the measurements have not been correctly processed or received.
(4) **Ubiquitous personal trainer.** This service provides the elderly with training programs and real-time feedback on his performance when practising specific exercises. It also generates alerts if bio-parameters are altered beyond safety limits. Additionally, the elders’ data may be stored through a cloud service for posterior analysis, benchmark and social evaluation. The visualization devices during the training session may include smartphones, projected interfaces or smart objects.
(5) **Ubiquitous gait freezing detector for Parkinson disease,** which provides an audio cue to unblock the pacer when he suffers a freezing episode. Real time intervention is important in this case. The sensors to monitor gait may be placed in different parts of the lower body and in shoes. In many cases, more than one sensor is needed, being data synchronized and processed in an external component of the sensor network.

**Table 1:** Model to analyse requirements of services.

Category	Characteristics	Description
(C.1) Sensor architecture	Sensor kit	Sensing hardware
	Sensor observations	Type of observation, which may be signal (signal strength, 3-axis acceleration, etc.) or feature level (location, fall detected, *etc.*)
	Sensor network type	The configuration ofthe sensing infrastructure: smartphone-bascd, body area network, infrastructure WSN, *etc.*
	Sensor kit / platform	Number of sensor kits that should be governed by the same platform.
(C.2) Consumer application needs	Data consumption mechanism	On demand, event-driven (condition-based and contract­based).
	Persistency needs	Need to store generated data.
(C.3) Messaging platform- consumer application	Information format	Description language (JSON, XML, datastream, etc.)
	Standardization	Need to pack data in a standard format (e.g. CAP, HL7, SNOMED...)
	Length	Number of bytes
	Expected output rate*	The average frequency for the platform to notify the consumer applications.
(C.4) Quality of service	Real time response needs	Hard (missing a deadline is a total system failure), firm (infrequent deadline misses are tolerable, but may degrade the systems quality of service, the usefulness of a result is zero after its deadline), soft (the usefulness of a result degrades al1er its deadline, thereby degrading the system's quality of service).
	Reliability needs	Tolerance to packet losses.
	Availability needs	The probability for a system to be operational at a given time
(C.5) Scalability	No. simultaneous consumers/producer	Number of simultaneous consumers that may be requiring data from a single producer.
	No. simultaneous producer/consumer	Number of users that may be simultaneously connected to a single consumer.
(C.6) Privacy and security	Privacy needs	Sensitive information has to be hidden to unauthorized users
	Security needs	Sensitive information has to be managed with security techniques: cryptography, signing, data integrity.

Note: * Low (f < 0.00028 Hz; T ≈ 1 h), medium (0.00028 Hz < f < 0.017 Hz; 1 h < T < 1 min), high (0.017 Hz < f < 1 Hz; 1 min < T < 1 s), very high (f > 1 Hz; T < 1 s).

Table 1 proposes a “model” to analyse these grass-roots service needs. In our approach, all these services will be coordinated by two types of components: a number of producers and one or more consumer applications/services. The former are sensors that push information related to the services towards the WoT-based platform. The later, which contain the service logic, are a processes running some device (e.g., user device or server) that gets data from producers by using the functionalities of the WoT-based platform. The model considers different aspects, such as the sensor architecture (type of observations, sensing infrastructure, *etc.*), the features of the consumer side (type of applications consuming the acquired data, the data consumption mechanism or the need for persistence), the characteristics of the message formats between the platform and the consumers (language, standards, length. . . ), general aspects of the QoS that the service must provide (real time, availability and reliability needs) and scalability (in terms of average rate between consumers and producers and the other way round). Additionally, services that deal with health data are considered as highly sensitive by the current laws on privacy and data management (e.g., see art. 8 of [23] or [24]), thus some requirements may focus on getting sufficient protection in terms of privacy and security. For the model, we assume that the sensors generate complete data, with acceptable quality for health support.

As the reader will notice, each of these services articulates different value proposals and differs in their target users. For example, services 3–5 are providing services to the elderlies themselves, while services 1–2 focus on external users (carers, members of the family network, *etc.*). Some of the services rely on sensors that are able to forward the already processed features to the platform (e.g., embedded fall detection), while others need the platform to include processing modules to detect events (e.g., location changes). In all cases, the installation of the WoT-based platform would be done in a smartphone or portable device, allowing its easy deployment in whatever environment.

Taking the model in Table 1 into consideration, Table 2 analyzes the services listed above by identifying their main requirements. With this analysis in mind, next section underlines the main functional and non-functional requirements that will guide the design of our solution.

### 3.2. Requirements for a Versatile WoT-Based Open Platform

The service analysis in Section 3.1 allows us to better identify the functional (focused on the system behavior) and non-functional (focused on the system constraints) requirements that should drive the design of a WoT-based open platform enabling numerous services that consider health and behavior data. We following list the four most relevant ones, which have been related to the analysis collected in Table 1 and mapped within the design principles described in Section 4.1.

*(R.a) Management of heterogeneous embedded devices and consumer applications*. It has been proved the feasibility of the WoT to handle a large heterogeneity of sensor and actuator devices that produce real-world related information (C.1). One of the objective of a WoT-based platform should reach a *one-fit-all* solution to support such diversity of technologies. However, while interoperability techniques are being standardized, WoT-base platforms should implements a variety of protocols to support most representative technologies. Nevertheless, the experimental use of ongoing standards this type of platforms (C.3) could provide valuable feedback to consortia working on standardization and harmonization of communications between sensor and actuator technologies.

On the other hand, many different types of consumer applications has to be supported. Their requirements are disparate in terms of Quality of Service (C.4), data consumption mechanisms, data persistence, security (C.6), *etc.* Thus, a WoT-base platform should implement a common solution that satisfies particular requirements of context data consumption.

*(R.b) Discovery and search mechanisms*. A WoT-based platform needs to be aware of the smart things composing smart environments (C.1): their identity and the resources they provide should be discovered as soon as they are deployed on a smart environment. Besides, efficient discovery mechanisms and protocols have to be designed keeping in mind the high dynamicity of smart environments (sudden “death” or connections as well as malfunctions of hardware components), that is usually produced by mobile devices and other constrained devices (e.g., those battery powered).

Data describing profiles of smart things (capabilities, resources, ownership and other information of interest) should be collected in a logical and structured way in order to be stored in a *knowledge base*. This knowledge base could be use by search engines in order to provide useful information about the current situation of a smart environment to entities that want to use their available resources somehow. The scalability and organization schema of the knowledge base will be an important factor to get quick and accurate results when searching information of available resources and their interrelationships (C.2 and C.5).

*(R.c) Easy definition of interfaces to access real-world services*. Every WoT-based platform must provide a uniform and open interface that allows consumer entities to access to resources of a smart environment though well-known Web technologies. Those interfaces have to be hardware-agnostic regardless the technological platform characterizing the smart things.

Security and privacy issues have to be taken into account when accessing resources (C.6). The security level and type of techniques to be applied for each resource will be defined according to sensitivity of the interchanged data.

*(R.d) Resource composition and orchestration*. Let us consider a WoT-based platform accomplising the R.b, then it could be able to provide functionalities to compose and orchestrate resources offered by disparate smart things (C.2). This could be an optional but valuable feature. The concept of *mashup* encompasses this idea, among others. In general, *mashups* can be seen as a complex resource that hides the composition and orchestration of a set of atomic resources with different goals.

Finally, it is necessary to specify a language to describe workflows according to rules that orchestrate the invocations to resources as well as the data interchanged in each step of the workflow.

## 4. The Micro Web of Things Open Platform

The above-mentioned requirements have driven the design decisions of the platform that is following described: the Micro Web of Things Open Platform, aka *µ*WoTOP. Willing to provide a holistic solution both for mobile health monitoring, wellness and Ambient Home Care Services, we hereby propose an architecture that can be customized in different application settings making a minimum extra effort. In particular, we have worked on our existing proposals [14] to adapt them to the requirements that a health scenario may require. The system architecture is supported by three key elements performing different roles:

- *Wireless biometric sensors and their data fusion components*: A heterogeneous set of biometric sensors (e.g., heart monitor, accelerometer, body thermometer), some of which are capable of performing a previous preprocessing of sensors readings and communicate their result to other architectural elements.
- *µWoTOP Gateways*: Their major role is to setup an event-driven message bus collecting health events detected by the wearables and transmitting urgent notifications to those entities interested in such events (e.g., medical staff at a hospital or assistants at a residence). *µ*WoTOP Gateways (or Smart Gateways) can run on two kinds of devices: infrastructure devices (*i.e.*, fixed devices), or mobile user devices (e.g., smartphones or tablets enabled as gateways).
- *Consumer applications*: These are pieces of software that run on user devices, both mobile and fixed (e.g., mini PCs or smart TVs). They have to implement a module to use the services offered by the platform, e.g., the eventing service.

**Table 2:** Analyzing different service scenarios.

		Gait-freezing detector	Personal trainer	Daily medical check-ups	Routines anulyzcr	Fall/faint detector
	**Sensor kit**	Shimmer	Fitbit	Scale	Smartphonc	Bioharncss
				Tesiometer		
			Ear pulse sensor	Aura	Pressure mats	Shimmer
				User input		
**Sensor Architecture**	**Observation**	Foot acceleration signal	Activity	Weight	Location, activity	Skin temp., heart rate
				Blood pressure		
			Pulse	Sleep quality	On/off	Fall alert
				User input		
	**Network type**	BAN	BAN	Infrastructure	Mobile	BAN
					Infrastructure	
	**Sensor kit/Platform**	1	1	1	1	100
**Consumer app. needs**	**User/Type**	Patient/mobile app.	Trainee/web service, mobile app.	Doctor/web service	Carer/web service, mobile app.	Carer/mobile app.
	**Consumption mode**	Condition	Contract, condition	Contract	Contract, condition	Event-based
	**Persistency**	Cloud event logs	Cloud sessionlogs	Cloud sessionlogs	Cloud daily logs	Local event log
**Messaging**	**Format**	JSON	XML	XML	Datastremning	JSON
	**Standard**	HL7	N/A	HL7, SNOMED	N/A	CAP
	**Length**	Short	Medium	Short	Short	Short
	**Output rate (P->C)**	Low	High	Low	High/very high	Low
**QoS**	**Real time**	Hard	Firm	Soft (repeatable measure.)	Firm	Hard
	**Reliability**	High	Medium (packet losses affect quality)	Low	Medium (packet losses affect quality)	High
	**Availability**	High	Medium	Low	Medium	High
**Scalability**	**Consumers/Producer**	1/1	Some/1	1/1	10/1	10/1
	**Producers/Consumer**	1/1	1/1	Many/1	1/1	100/1
**Privacy/Security**	**Privacy**	High	Medium	High	High	Low
	**Security**	High	Low	High	High	Low

We following detail the design principles and resulting architecture for *µ*WoTOP.

### 4.1. Design Principles

The design of the *micro* Web of Things Open Platform (*µ*WoTOP) is based on an open platform that was initially specified for unconstrained devices, e.g., PCs or servers. The major pillar of the *µ*WoTOP architecture is its open nature that provides developers with tools to allow them to build mashups of resources by means of defining interactions among entities in a smart environment. Specifically two different roles are defined: (a) producers of information and (b) consumers of information. These resources are representations of entities and their capabilities that are available in a smart environment, which converge into the *µ*WoTOP through essential functionalities provided by it. The architectural model of the *µ*WoTOP is based on six design principles which take into account the system requirements gathered in Section 3.2. Essentially, *µ*WoTOP has been designed to:

(1) Facilitate and enhance the connectivity among heterogeneous IoT-based networks composed of embedded sensor and actuator devices; this design principle guarantees the requirement *R.a*. implementing both standard and proprietary protocol stacks that cannot be mapped directly into IP/TCP. The interconnection of these networks is coordinated through Smart Gateways, which are properly synchronized in order to share their resources. Every Smart Gateway will implement the IP/TCP protocol stack by default and specific protocols; those protocols could be standardized or privative and they will be focused on supporting embedded devices plugged directly to the Smart Gateway.
(2) Discover, configure and manage large ecosystems composed of heterogeneous smart things. This feature is tightly related to the requirements *R.a*, *R.b* and *R.c*. Typically, the concept of smart thing is defined as a set of sensor and actuator devices that, in conjunction, can reach common goals, e.g., to infer complex context information by gathering sensor measurements. Additionally, the Smart Gateways offer a mechanism that facilitates the interaction of smart things each other, as well as among smart things and external entities, by means of well-defined interfaces.
(3) Offer all available resources (e.g., sensor, actuators and processes) by means of Web technologies according to the Web of Thing paradigm. For this purpose, a set of standardized, uniform and dynamic interfaces, based on the REST architectural style [25,26], will be provided; this characteristic potentially resolves the requirement *R.c*. The REST API defined to access those resources will be completely public according to the “open” nature of the *µ*WoTOP, *i.e.*, HTTP methods, URIs, format of messages and responses will be well documented and accessible for everybody with an Internet connection.
(4) Use standards and recommendations (R.a) to define information models managed by the *µ*WoTOP in order to dispatch messages that are understandable by as many applications as possible, e.g., Common Alert Protocol (CAP) for the definition of emergency notifications related to health events. This design principle should facilitate the achievement of all of the requirements previously described as the use of standard protocols and documents makes easy the integration of *µ*WoTOP with applications already on the market.
(5) Support common message delivery modes in order to be able to deliver information to clients as well as to interrelate resources modelling *mashups* according to the interaction requirements of each scenario. Two different message delivery modes are supported: (i) on-demand and (ii) event-driven. Both message delivery modes can be accessed using the REST API mentioned in the second design principle. This specific point solves the requirement *R.d*. Moreover, the achievement of the requirement *R.c* facilitates this requirement.
(6) Provide mechanisms for guaranteeing the security, mostly confidentiality and integrity, of sensitive information according to the requirements of particular scenarios, e.g., events carrying health information from a patient in a hospital with a venereal disease. This design principle should be transversal to all of the requirements previously described taking place in one sense or another depending on the sensitivity of specific scenarios.

The general purpose behind the design principles described above is to provide a wide range of features and tools to conduct the building and use of any smart space based on heterogeneous ecosystems composed of hundreds, even thousands, of smart things. This facilitates the prototyping of complex applications and services according to the functionalities offered by the smart things deployed on smart environments; those functionalities are usually related to real-world services, *i.e.*, services that bind physical phenomena with virtual entities. Moreover, the development environment provided by *µ*WoTOP allows easily reusing and sharing already deployed resources as much as possible. Thus, different developers could create mashups of real-world services sharing the same set of resources. Additionally, this feature provides a variety of interaction mechanisms to communicate applications with smart things.

It is also needed to deal with very strict non-functional requirements: the necessity of deploying on constraint devices as well as the support of an acceptable scalability level among others. Those requirements represent the major challenges in the implementation and deployment of the *µ*WoTOP architecture; they will be key factors in the search of a trade-off between pervasiveness and performance of the *µ*WoTOP-based systems. The next Section specifies the architecture of the *µ*WoTOP.

### 4.2. Architecture Overview

**Figure 1:**
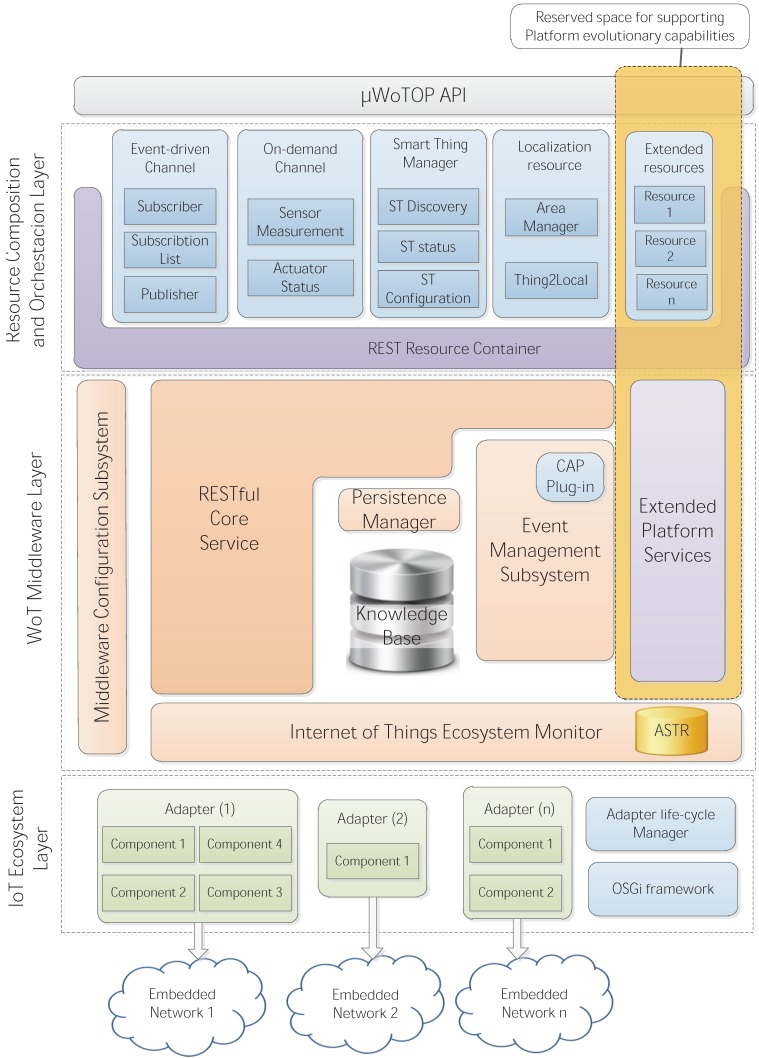
Conceptual diagram of the *µ*WoTOP’s architecture.

*µ*WoTOP was specified according to a layered architecture organized in three layers that are interconnected by means of well-defined interfaces. The next paragraphs explain, from a bottom-up perspective, the main features of each one of these three layers as well as their major components and interfaces as shown in Figure 1.

**(i) *Internet of Things Ecosystem Layer***: The objective of this layer is to deal with an ecosystem of sensor and actuator devices in order to be modeled as information resources following the principles of the REST architectural style. Therefore, this layer can be considered as the major interface to the real world in which users coexists with embedded (and hopefully invisible) sensor and actuator devices. The major functionality of this layer is to identify all the potential resources, which are usually mapped on sensors and actuators, classify them according to their typology and, finally, announce them to the upper layers in a hardware agnostic fashion. The components in charge of performing this process are called adapters. The adapters wrap drivers for every technological platform connected directly to a *µ*WoTOP Smart Gateway, which allows discovering every sensor and actuator populating the environment. The schema of adapters was designed to provide to the *µ*WoTOP Smart Gateways with capabilities for plugging any device according to a Plug&Play paradigm. The lifecycle is scheduled by the Adapter Execution Manager which is based on the OSGi framework. Therefore, every adapter is implemented as an OSGi bundle that is stored in an OSGi Bundle Repository (OBR); when a new device is connected to the *µ*WoTOP Smart Gateway a bundle implementing the correspond adapter is installed and initiated. From that moment, the device capabilities will be discovered by the Internet of Things Ecosystem Layer. Afterwards, those capabilities are announced to the components integrated into the Web of Things Middleware Layer, which will transform them into a more abstract information model according to the principles of the REST architectural style.

**Figure 2:**
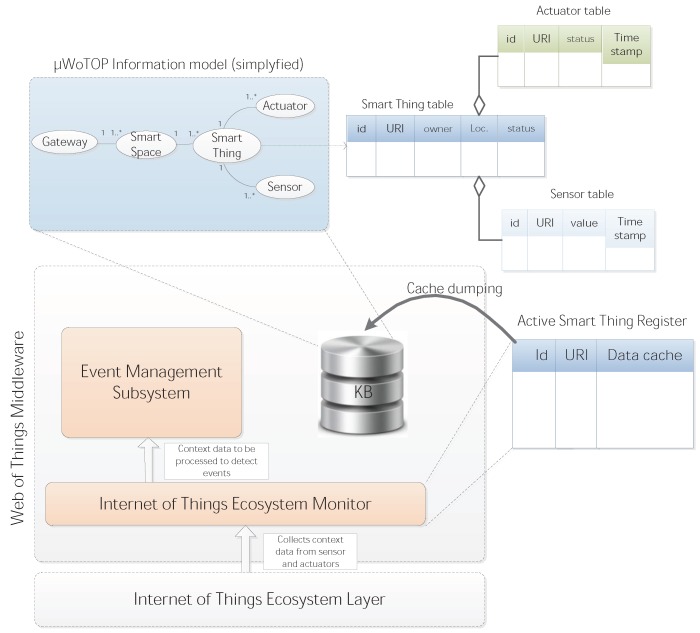
Procedure for collecting context information data generated in the Internet of Things Ecosystem Layer.

**(ii) *Web of Things Middleware Layer***: The major role of this layer is to provide a set of functionalities that allows designers and developers of real-world services to design, develop and deploy smart spaces following a complex schema of interactions among the smart object belonging to them. The major components of this layer are next detailed:

**(ii.a) Internet of Things Ecosystem Monitor**: This component aims at controlling, in an organized way, the data streams coming from the adapters deployed on the Internet of Things Ecosystem Layer. The two major tasks of this layer are: (i) to collect the list of device capabilities (sensors, actuators and other context information sources) discovered by the lower layer and map them into an information model for managing smart spaces; (ii) to manage a bidirectional data flow consisting of gathering data generated by context information sources (sensors and business processes) as well as to forward messages to actuators. In order to facilitate these processes, a register, called Active Smart Thing Register (ASTR), is created and maintained with a complete list of resources hierarchically organized according to the information model for smart spaces. The ASTR defines several types of entries whose relations and multiplicity are specified in the mentioned information model. These entries are used not only to identify a list of available resources in the smart space and their access points but also to keep a cache with the most recent states of every resource, e.g., the values of sensors or status of actuators. These caches are periodically dumped into a Knowledge Base (KB) which stores the information managed in the smart space at any point. That information can be retrieved by external entities which are allowed to access it. This process is shown in the Figure 2.

**(ii.b) Event Management Subsystem**: This component implements the engine that enables the platform to manage an asynchronous communication mode that is a crucial requirement for any event-driven mechanism. Thus, this subsystem is involved in processes for notifying events to external entities (mostly applications and services) which are subscribed to them. The event-driven mechanisms implemented by the Event Management Subsystem are two:

- *condition-based*: this mechanism is used to dispatch events when context information, which is generated by sensors and other processes, matches with some condition set up by consumer entities. For example, a simple scenario working through this event-driven mechanism could be based on an ambient intelligent system that sets up a condition as follows: a presence of user X is detected in the living room AND the ambient temperature of the living room drops below 21 °C.

- *contract-based*: this mechanism dispatches specific data to consumer entities when an internal timer event is triggered. By means of this mechanism, data are dispatched periodically as long as the cache in ASTR contains updated values or until the contract-based subscription is finished and, consequently, the internal timer is stopped. A common use case of this type of event-driven mechanism could be a localization system requiring gathering updated localization information from a mobile node (coordinates) every 2 s, for 30 min. The abovementioned event-driven mechanisms have to be initiated by means of specific types of subscriptions that demand an explicit interest in being notified about the occurrence of specific types of events. A subscription is defined by the following parameters:

*Producer id* (string): it indicates the URI of the event producer managed by the *µ*WoTOP. *Consumer id* (string): it indicates the URI of the event consumer. Basically, it identifies a listener entity which is waiting for events. *Event type* (number): it specifies the event-driven mechanism to be used for the particular subscription: conditional-based or contract-based. *Subscription start time* (number): timestamp indicating when the subscription starts to be valid (in Unix Time format). *Subscription end time* (number): timestamp indicating when the subscription is no longer valid (in Unix Time format). *Payload format* (string): it specifies the description language which will define the event payload. By default, JSON is used. *Security* (string): it indicates if security mechanisms will be used when an event has to be forwarded to a consumer. By default, no security technique is used.

Condition-based subscriptions are defined by the following additional parameter: *Event occurrence conditions* (string): it defines one or more conditions specifying the occurrence of an event.

Contract-based subscriptions are defined by the following additional parameter: *Sampling period* (number): it indicates the frequency of data forwarding as long as updated data is available in the cache of the ASTR.

**Figure 3:**
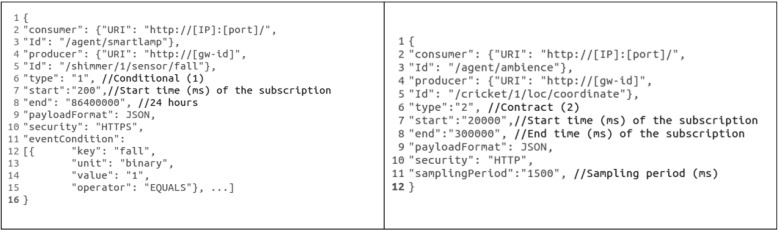
Two subscriptions managed by the Event Management Subsystem. On the right, a contract-based subscription; on the left, a condition-based subscription.

Subscriptions managed by the Event Management Subsystem are all defined in JSON language. Fig. 3; shows two documents defining the two types of subscriptions described above.

Once the consumer entity has sent a subscription, it must open a Webhook which enables it to listen to the event occurrence it subscribed to. A Webhook is a specific kind of asynchronous programming technique that is based on defining HTTP callbacks (characterized basically by a HTTP method, a URI and an accepted media type) to gather information when some other processes invoke it. The Event Management Subsystem instantiates Webhooks according to the parameters indicated in every subscription in order to forward events to the right consumer application or service. It is important to highlight that the Event Management Subsystem allows securing data transmission to Webhooks according to the security requirements of the specific scenario. In order to accomplish with the REST paradigm, the *µ*WoTOP offers HTTPS over SSL to provide security mechanisms. Basically, security aspects that are guaranteed through HTTPS are authenticity, integrity and confidentiality of data exchanged between consumer entities and the *µ*WoTOP. This is enough for the most of applications that manage sensitive information (e.g., ehealth, home security or social applications).

**Figure 4:**
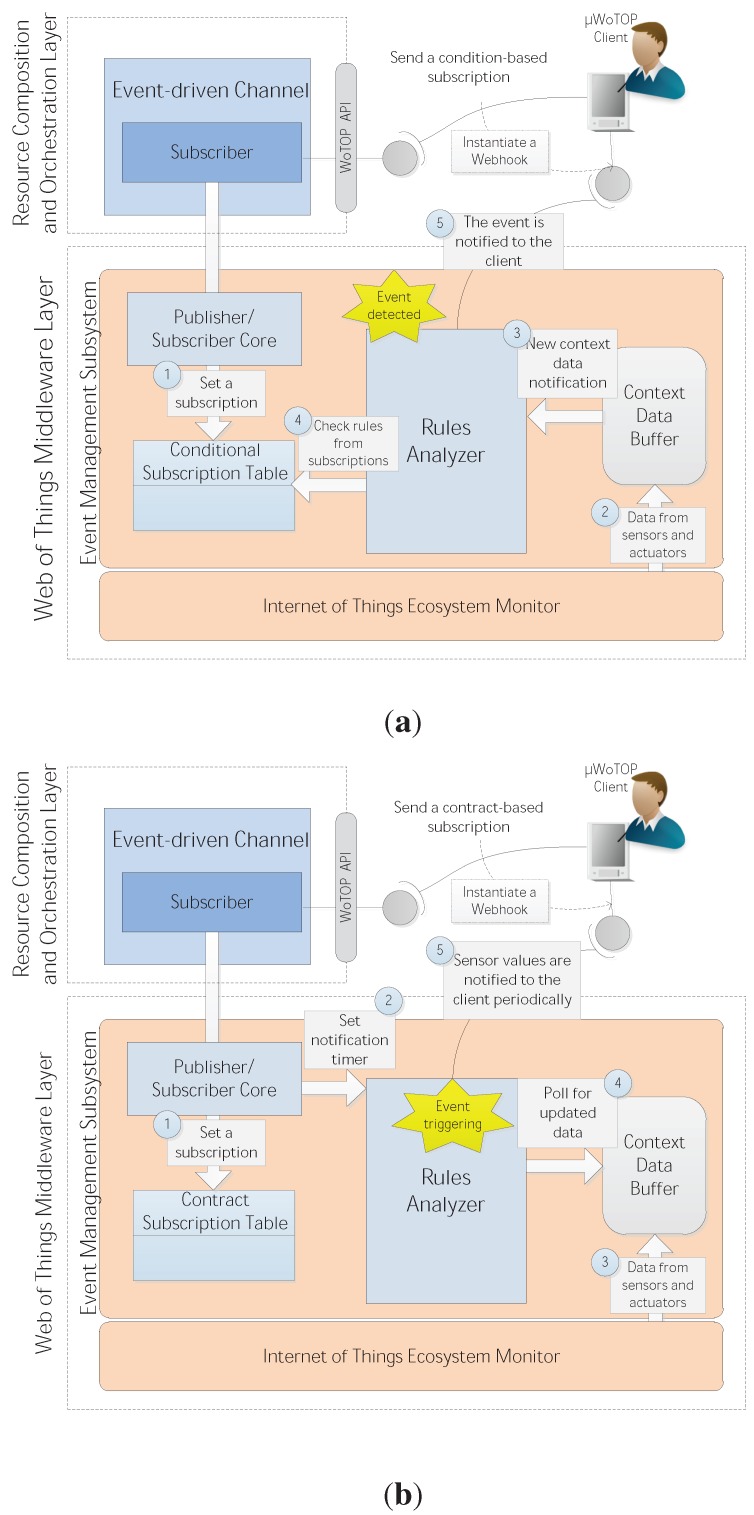
The two event-driven communication techniques supported by *µ*WoTOP: (**a**) condition-based; (**b**) contract-based.

**Figure 5:**
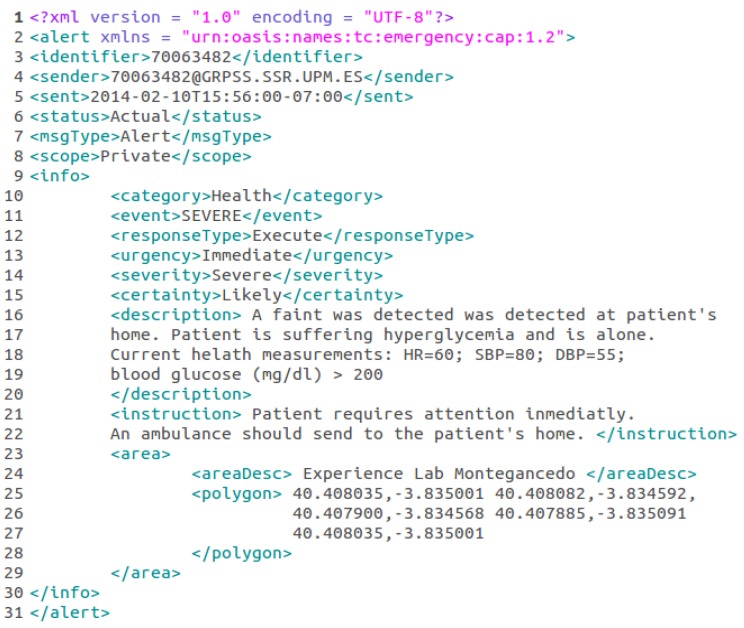
A CAP message describing a health alert related to physiological parameters of a user of a smart gym.

Additionally, the Event Management Subsystem includes a mechanism to use plugins that optionally puts events into standardized formats just before dispatching them to the consumer applications. This feature makes *µ*WoTOP compatible with alerting technologies that manage warnings and emergencies with different critical nature. The current version of *µ*WoTOP implements a plugin that provides support to define and interpret the Common Alert Protocol (CAP). CAP is a standardized description language based on XML, which was designed to integrate seamlessly different public alerting systems. The International Telecommunication Union, Telecommunication Standardization Sector (ITU-T) approved CAP as the Recommendation X.1303. Currently, some companies and public organizations all over the world have deployed CAP-based system (e.g., United States Department of Homeland Security, Public Alerting and Notification (Canada) or the Emergency Management Australia). The CAP message structure provides detailed information about any alert. The CAP recommendation specifies several fields to define an alert, some of them are mandatory and other optional. For example, the mandatory field message type (msgType) denotes the nature of the alert, and the optional field info (info) defines the container for all component parts of the info sub-element of the alert message that provide additional information, e.g., area of occurrence (area), urgency level (urgency) or description of the event (description). Figure 5 shows an example of a CAP message generated by the Event Management Subsystem that would be encapsulated by an event occurred in an eHealth system.

**(ii.c) Middleware environment configuration component**: This component enables to set up execution parameters of the middleware layer. In order to properly configure the middleware execution environment, some important particular characteristics of the underlying infrastructure has to be taken into account to optimize the performance and scalability of the platform. Some typical parameters that can be configured are the maximum number of simultaneous execution threads attending client requests, the maximum number of subscriptions, or the address and credentials to access to the knowledge base. **(ii.d) *Middleware Core***: This component is an essential key in the *µ*WoTOP architecture. It is in charge of coordinating the initialization sequence of the platform, e.g., signaling to the lower layer for start listening for devices connections, to establish a connection with the knowledge base, or to load the needed component to set up accessible REST end-points to the platform services. The latter is especially important since it allows exposing the major functionalities of the platform. Additionally, it provides a REST framework to deploy extra components that implement new functionalities and services extending the *µ*WoTOP capabilities. Those components are deployed on the upper layer called Resource Composition and Orchestration Layer.

**(iii) *Resource Composition and Orchestration Layer***: As mentioned above, this layer is used to deploy components enabling *µ*WoTOP to offer platform services as well as additional services and functionalities in a RESTful fashion. The lifecycle of the components deployed on this layer is controlled by a resource container. This lifecycle is planned in five phases: (1) loading; (2) starting; (3) running; (4) destroying; and (5) stopping. The first (loading) and the fourth (destroying) phases allocate and free up memory for the necessary structures for the operation of the component, respectively. The second (starting) and the fifth (stopping) phases are defined for opening and closing the REST end-points that enable accessing to resources implemented by the components. During the third phase (running) resources are accessible, and provide services and functionalities according to the design features. Those resources will be queried by using HTTP methods (*i.e.*, GET, PUT, POST, OPTIONS or DELETE) on the URI that identifies the resource uniquely.

The fundamental platform services provided by *µ*WoTOP are offered by means of several resources that are deployed on the Resource Composition and Orchestration Layer by default. Those resources provide essential functionalities, among others, the communication modes supported by the platform: (i) event-driven and (ii) on-demand. These communication modes are provided by the Event-driven channel and On-demand channel resources. Additionally, functionalities to register/unregister smart things and update their states, typically offered by the Internet of Things Monitoring Component through the Internet of Things Ecosystem Subsytem, are provided by means of the Smart Thing Manager resource. This resource allows autonomous devices to use the platform services without having to be plugged directly to a smart gateway. Moreover, a Localization resource is deployed which provides fundamental localization services, e.g., identification of areas in which a target is localized. The integration of a localization service into the Resource Composition and Orchestration Layer was motivated due to the fact that a very common feature of many smart spaces is the location-awareness, e.g., customized eHealth spaces for monitoring patients outside. Finally, the Resource Composition and Orchestration Layer allocates part of the resource container for deploying additional resources that are developed with the purpose of extending the essential platform services. This feature provides the platform with evolutionary and adaptability capabilities, which enable it to tackle different scenarios according to the open nature of the *µ*WoTOP. The idea behind this point is to create a scene of developers that contribute to a common repository of components of resources. The following section explains the procedures that lead the development and deployment of software to add functionalities to the *µ*WoTOP.

### 4.3. Development Guide to Build Smart Spaces Using µWoTOP

In accordance with its open nature, the *µ*WoTOP provides developers with extension mechanisms that are focused on using evolutionary capabilities of its architecture. This mechanism is composed of procedures that are documented, step by step, in order to support to the developers who need to contribute to modify somehow the *µ*WoTOP architecture for a specific domain. The three extension areas that can be tackled by means of the above mentioned mechanisms are the following: *(i)* development of adapters for connecting new sensor and actuator devices to the smart gateway running *µ*WoTOP; *(ii)* development of resource components to extends the essential resources and offer new functionalities to the platform clients; *(iii)* development of business logic for client applications to access the *µ*WoTOP’s services. The following paragraphs describe a step-by-step guide on how to develop a whole smart space using the *µ*WoTOP architecture.

#### 4.3.1. Development of software to connect additional sensor and actuator devices to a smart gateway

**Figure 6:**
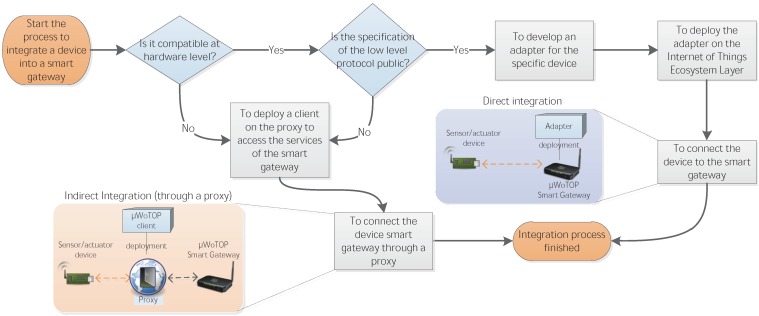
Integration process to connect sensor and actuator devices to the smart gateway running *µ*WoTOP.

As explained in the previous section, the *µ*WoTOP’s architecture offers two different methods to connect sensor and actuator devices to smart gateways running *µ*WoTOP (see Figure 6). The first method consists of developing adapters to enable a direct connection of devices to a smart gateway. This connection will be based on different hardware connectors and low level protocols (e.g., Wifi, Bluetooth or USB). The major requirements to carry out this method are twofold. On the one hand, the smart gateway has to be compatible at hardware level to connect physically sensor and actuator devices. On the other hand, developers of adapters need to be knowledgeable about the low level communication protocol of the devices to be connected in order to get messages generated by each device. As commented in Subsection 4.1, adapters are implementations of OSGi bundles which are stored in an OBR. Every adapter must set up an interface with the Internet of Things Ecosystem Monitor component of the middleware layer, in order to announce every device newly connected to a smart gateway with the purpose of keeping updated the ASTR of the Internet of Things Monitoring component. From this time on, the WoTOP will be able to handle a bidirectional data flow with all those devices connected directly to a smart gateway. The alternative method to connect devices to a smart gateway is to use the Smart Thing Manager resource. Devices using this resource need to implement functionalities to access RESTful interface by themselves or through a proxy, in case they do not have capabilities to implement the necessary protocol stack. This protocol stack is based on TCP/IP, and on top of it, the HTTP protocol. The Smart Thing Manager resource also invokes the services of the Internet of Things Ecosystem Monitor component. Devices connected through this method keep an entry in the ASTR and, therefore, a bidirectional communication is enabled like those connected directly to the smart gateway.

#### 4.3.2. Development of resource components to extend platform services and functionalities

Another extension mechanism for *µ*WoTOP consists of carrying out the development and deployment of new resources on the Resource Composition and Orchestration Layer which are exposed as RESTful services. The exposition of these resources as RESTful services needs to define REST end-points defined by, at least, three parameters: *(1)* a meaningful and readable URI; *(2)* a HTTP method and; *(3)* a media type of the message payload. These resources are deployed on an allocated part of the component container called Ambient Intelligence Configuration. All components deployed on the component container, including the ones deployed on the assigned area, can reuse functionalities implemented by other resources or by components belonging to the Web of Thing Middleware Layer what allows composing and orchestrating complex services and workflows involving a number of sensors, actuators and processes.

#### 4.3.3. Development of client application and services consuming *µ*WoTOP’s services

Both user applications and background processes using *µ*WoTOP services have to implement a middleware layer that enables them to invoke seamlessly those services. This middleware layer sets up a virtual message bus that facilitates the access to the platform services using the two communication mechanisms supported by *µ*WoTOP: *(i)* event-driven and *(ii)* on-demand.

Some middleware libraries are provided in order to facilitate the programming of REST-based client applications according to the mentioned communication mechanisms. Through this middleware layer, developers can manage the whole life-cycle of an application utilizing the *µ*WoTOP resources. Every part of that life-cycle is especially approachable by any developer since the underlying technical complexity is abstracted by the middleware (e.g., protocols, message interchange, event listener instantiations, *etc.*). To develop a simple client application that sends subscriptions, consumes events and, eventually, removes subscriptions, the following steps are needed:

(1) Implement an event listener: The business logic of the application has to be implemented as a component (it could be a Plain Old Java Object, POJO) which will be in charge of handling future incoming events that are dispatched by smart gateways. If incoming events are related to a health emergency, they will attach information in CAP format, which has to be properly parsed to identify every field of the event.
(2) Create a subscription: The subscription has to define, among other parameters, the URI of the event generator, the URI of the event consumer and the rules to trigger events, *i.e.*, a threshold, for condition-based subscriptions, or a sampling time, for contract-based subscriptions.
(3) Send the subscription: The subscription previously defined has to be sent to a specific or any smart gateway of a domain, which will be registered in a subscription table.
(4) Load an event listener: Immediately after sending a subscription, an event listener associated to that subscription has to be loaded in order to start listening to every event dispatched by a smart gateway.
(5) Send an unsubscription: Finally, before the application is unloaded, any subscription previously done has to be removed. In order to fulfill this process unsubscription messages have to be sent to smart gateways indicating the code of the subscription to be removed.

**Figure 7:**
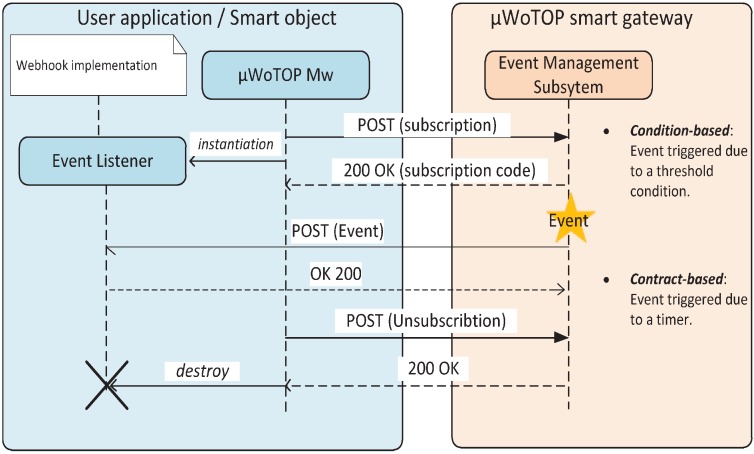
Message sequence showing lifecycle of an application based on the event-driven mechanism.

Figure 7 shows the life-cycle of applications developed according to the guide explained above. It requires an interchange of messages between user devices or smart object and one or more smart gateway. This message sequence is completely hidden to the users by the virtual message bus instantiated by the *µ*WoTOP middleware.

On the other hand, the on-demand mechanism is much simpler than the event-driven; it is usually used to perform synchronous request for context information to the smart gateway, but also to interact with the environment by starting an action (e.g., through an actuator or modifying an execution parameter). According to the REST paradigm, GET and PUT HTTP messages will be sent during this communication mode: the former to obtain context information, and the latter to cause an action over the environment.

## 5. Case Study: A Healthcare Monitoring System to Promote Independent Living

### 5.1. Motivation Scenario

As previously said, WoTOP has been designed with the objective of serving as basis to deliver personal health, wellness and ambient assisted living services. In particular, the purpose of the motivation scenario is to provide an integrated healthcare system promoting the independent living of those elderly who are considered outpatients associated to a specific clinical service, living at their respective homes or at a residential care place. The major functionalities offered by the system are twofold:

(1) To detect and notify risky situations for the outpatient’s health (e.g., fall and faints, abnormal cardiac rythm, *etc.*), in which the person may need the support of a doctor and, if necessary, a quick intervention coordinated by an emergency center.
(2) To monitor and gather vital signs and other health information of the outpatient in order to populate a Knowledge Base (KB) that could be processed to perform a quick diagnosis of illnesses or detect bad habits.

**Figure 8:**
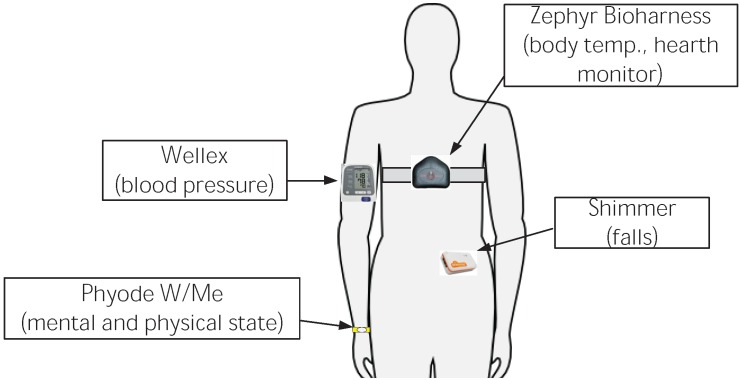
Wearable devices composing the Body Area Network (BAN) used in this case study.

Apart from the outpatient, there are other users involved on this scenario: outpatient’s relatives as well as medical staff associated to a clinic, who will be looking after the outpatient remotely. The infrastructure deployed on this scenario is very diverse, including from biometric sensors to personal devices. A classification of the elements of this infrastructure is listed below:

- *Fixed infrastructure*: The major element that encompasses this category is the Smart Gateway which is a non-intrusive and constrained device that can be easily integrated in the user’s environment. This devices could be just smartphones, tablets or mini PCs, based on the Android operating system, which run a *µ*WoTOP instance on it. The two main functionalities of the Smart Gateway deployed on the proposed scenario are, on the one hand, to coordinate event-driven communications among entities of the scenario and, on other hand, to manage the persistence of health data stored in KBs for subsequent analysis of the outpatient’s health state. Additionally, the fixed infrastructure is also composed of some auxiliary smart things that provide functionalities to adapt the patient’s environment depending on the user context (e.g., a smart lamp to set up the color and intensity of ambient light in case of detecting a complicated situation with regard to the patient’s health).
- *Body Area Network* (BAN) composed of a variety of wearable devices, which must be worn by outpatients and that measure some biometric parameters related to the outpatient’s health (see Figure 8). All those gathered measurements are dispatched to the Smart Gateway which detects the occurrence of events and store the data in a KB. Specifically for the considered scenario, the mentioned BAN is composed of the following biometric sensors (The wireless communication technology of all these sensor devices is based on Bluetooth.):

(a) *Biometric chest belt* (Zephyr Bioharness-like): This belt is equipped with a number of biometric sensors. Two of them are used in this scenario: hearth rate monitor and body temperature. This device usually transmits sensor measurements towards *µ*WoTOP with a period of 1 s by setting up a contract-based subscription. Missing measurements may degrade the information about the patient, e.g., in case of heart failure, data are critical.
(b) *Blood pressure wristband* (Wellex-like): This wristband is focused on measuring the blood pressure of the outpatient. This device usually transmits a blood pressure measure with a period of 5 min, also through a contract-based subscription. The same as in the previous case, the lose of measurements causes information degrading, but real time requirements are not so strict.
(c) *Accelerometer* (Shimmer-like): This accelerometer is attached to the belt of the outpatient. Basically, its objective is to monitor movements of the outpatient in order to detect accurately whether the outpatient has suffered a fall. The detection algorithm is implemented in the sensor. This device only sends messages when a fall has been detected. Thus, those messages can be considered as events themselves that are dispatched through condition-based subscriptions. In this case, real time performance is obviously a must.
(d) *Mood ring* (Phyode W/Me Wristband-like): This wristband is equipped with a life spectrum analyzer (LSA) that considers the body as a whole system. This sensor can measure the mental and physical state of an outpatient by analysing his ECG and breathing. This sensor needs a proactive collaboration from the outpatient who should put a finger on the LSA analyzer. This sensor only sends data towards *µ*WoTOP when outpatients carry out a measure by themselves. *µ*WoTOP forwards these data to consumers according to condition-based subscriptions. The user may repeat the measurement in case it is lost, thus its real-time requirement are soft.

(3) User devices, mainly smartphones and tablets, that are managed by outpatient’s relatives and medical staff associated to the system. In the proposed scenario, user devices run applications and services that can subscribe to the events occurring in the scenario as well as getting health data of the outpatient’s on demand through the persistence services provided by the Smart Gateway. Then, the retrieved data are processed in order to find some pattern that could facilitate the diagnostic of illnesses or bad habits in the outpatient.

**Figure 9:**
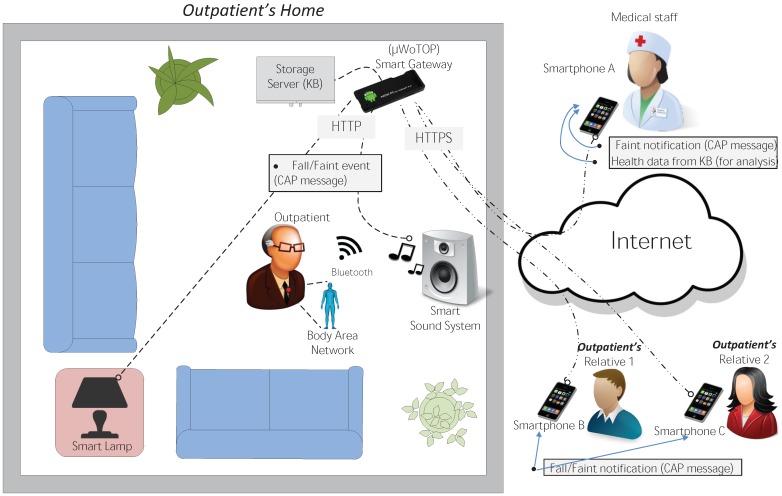
Motivation scenario: integral healthcare system for promoting independent living of the elderly.

The infrastructure described above is deployed principally on the outpatient’s home (see Figure 9) or, alternatively, on a medium-size residential care place. Since Smart Gateways are relatively resource-constrained devices, more than one could be deployed, depending on the scalability requirements of the particular scenario. In this case study we used a only one device (an Android-based smartphone) working as a Smart Gateway in order to evaluate maximum capabilities of a single Smart Gateway in terms of performance and scalability (specific features of the device used for testing are explained in Section 6). The BAN to be worn by the outpatient’s was synchronized with that Smart Gateway, sending sensor measurements periodically or asynchronously using their wireless communication capabilities.

In order to detect falls and faints, we implemented two algorithms that run in a distributed way: one for fall detection and another for faint detection. Those algorithms use context information provided by wearable of the outpatient’s BAN. Implementation details and other infrastructure issues involved on the execution of those algorithms are described in the next Section.

When an event is inferred by one of the mentioned algorithms, the Smart Gateway processes and dispatches them to one or more agents running on different entities deployed on the scenario. Apart from those agents, other agents will be subscribed to other type of events in order to consume data asynchronously, as well as gathering data on demand. Three types of agents are considered for the motivation scenario:

(a) Notification agents running on the user devices that belong to outpatient’s relatives. These agents are listening for fall and faint events in order to reveal to outpatient’s relatives the occurrence of a likely dangerous situation.
(b) Health monitoring agents running on the user devices that belong to medical staff associated to a clinic. These agents are listening for faint events in order to notify to medical staff the occurrence of a faint suffered by an outpatient that could indicate a critical health situation. The information provided to the doctor allows him to proceed adequately according to the specific emergency.
(c) Ambient adapter agents running on smart things that are deployed on the outpatient’s home or residential care places. These agents are aimed at receiving fall and faint events in order to adapt the user’s environment to calm him down when he is under a high stressing situation.

As mentioned in Section 4.2, alert-based events managed by *µ*WoTOP encapsulates CAP documents describing critical context information. In that way, health events generated in the proposed scenario will transport CAP documents describing essential details of outpatients’ context, e.g., where the outpatient is exactly localized and his current vital signs. The information interchanged among entities deployed on this scenario is, mostly, conducted by means of event-driven mechanisms. Table 3 shows the relationship among those entities (producers and consumers of events) including the input data that generate events and the type of event that is dispatched to the consumer entity.

**Table 3:** Relationship between event producers and event consumers in the motivation scenario.

Event Producer	Data Input	Generated Event	Event Consumer
Fall detector algorithm (Shimmer)	3-axis accelerometer event measurements pressure sensor	Fall event	Notilicacion agents Ambient adapter agents
Faint detector algorithm	Blood pressure sensor measurements Hearth monitor measurements	Faint event	Notification agents Health monitoring agents Ambient adapter agents

**Figure 10:**
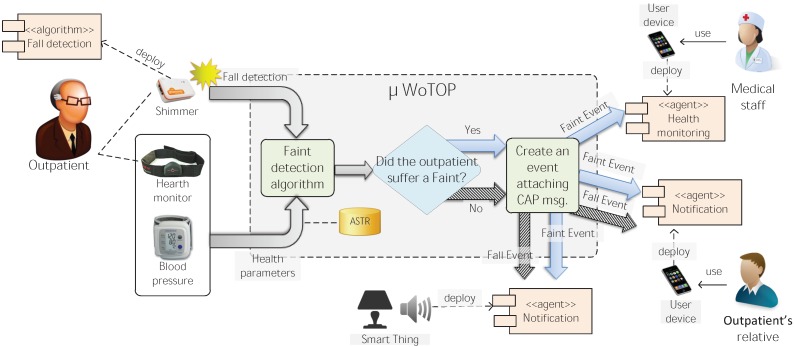
Motivation scenario: integral healthcare system for promoting independent living of the elderly.

When a Smart Gateway gets a fall event from a Shimmer the system starts a workflow that can follow different paths depending on the analysis of the context information (specification details of the algorithm are explained in the next Section). For example, if a fall is detected, with no additional complication, then an event encapsulating a CAP message explaining details of the situation is dispatched to both notification agents (deployed on user devices of outpatient’s relatives) and ambient agents (deployed on smart things). The former is intended to assist the outpatient as soon as possible without medical support, while the latter is aimed at adapting the ambient of the place in order to mitigate stressing situations for the outpatient. On the other hand, if a fall is detected together with some additional problem that could have produce a faint (e.g., low blood pressure) then an event encapsulating a CAP message is dispatched to both medical staff of the associated clinic and outpatient’s relatives, as well as to an a smart thing deployed on the outpatient’s environment. The two first notifications are intended to assist the outpatient efficiently and quickly according to the attendance protocol specified for that emergency and to notify to the outpatient’s relatives about that critical situation. As previously, the second is intended to trigger a mechanism that creates a relaxing ambient to mitigate the stress of the patient. Figure 10 shows the workflows described above.

From the decisional node inferring the occurrence of a faint (the diamond-shaped one), arrows filled with diagonal lines denote the instantiation and notification of a fall event encapsulating a CAP message. On the other hand, arrows filled with solid color indicate a path which instantiates and notifies a faint event encapsulating a CAP message that is specified by parameters describing the outpatient’s situation, *i.e.*, current health state, coordinates of the area, criticality of the situation, *etc.* In both of the previous paths, events are collected by smart objects deployed on the outpatient’s environment.

### 5.2. Biometric Sensors and Techniques to Detect Falls and Faints

In order to detect falls and faints, we use the information provided by a 3-axis accelerometer, a heart rate monitor and a blood pressure sensor. In particular, falls are detected by analysing the measurements of a 3-axis accelerometer embedded in a Shimmer device, which is attached to the patient’s belt. Faints, on the other hand, are considered as a special case of falls in which the vital signs of the patient are altered. To detect them, we use the data provided by a heart rate and a blood pressure sensors included in the Zephyr Bioharness chest belt and Wellnex bracelet, respectively. Apart from the embedded 3-axis accelerometer, the Shimmer device also includes a programmable microprocessor and a Bluetooth radio chip. Thanks to this, the fall detection algorithm can be programmed on the wearable device to detect anomalous peaks in the acceleration measurements (falls) and send a message through the Bluetooth link to the Smart Gateway in order to notify this event. This has the advantage that the complete raw data does not need to be transmitted continuously, which will extend the battery life of both the transmitting and the receiving devices (the wearable sensor and the *µ*WoTOP Gateway, in case it is mobile). However, the Shimmer device has low processing and memory capabilities, so a light algorithm must be used. In our case, the accelerometer signal is processed using a second-by-second scheme, taking the data gathered in a 1-s window. Furthermore, taking into account that 99% of the energy of the human body movement is contained below 15 Hz [27], the sampling frequency of the accelerometer is chosen to be 30 Hz (for each axis). The fall detection algorithm is based on the method described in [28] (with some differences).

When a fall is detected by the Shimmer device, the *µ*WoTOP Gateway receive the corresponding event notification through the Bluetooth connection and determine whether that fall event is just a fall or, on the contrary, corresponds to a faint situation (see Figure 10). To this end, the data provided by the Zephyr and Wellex devices worn by the patient are analyzed. Those devices measure health parameters of the patient and send the complete raw information through a Bluetooth connection to the *µ*WoTOP Gateway. Both devices are not programmable, so the signal is analysed externally in the *µ*WoTOP Gateway. For this purpose, we have developed a plugin to extend the *µ*WoTOP architecture that processes the data gathered from the Zephyr and Wellex when a fall event is detected in order to decide if the patient has fainted. Figure 10 shows the workflow of this process, from a fall is detected until the corresponding notification is dispatched to the agent listening for a specific event (fall or faint event). Agents consuming that events are running on user devices (smartphone or tablet) that are managed by medical staff or outpatient’s relatives. As commented above, other agents are deployed on smart things which are focused on alleviating stress level of outpatients while corresponding assistance is coordinated. The faint detection algorithm can be summarized as follows:

(1) If a fall event has been detected by the Shimmer sensor and received by the Gateway, the heart rate (HR), the systolic blood pressure (SBP) and the diastolic blood pressure (DBP) received from the Zephyr and Wellex devices at that moment are compared with their values 60 s before. Previous gathered readings are retrieved from corresponding temporary cache of the ASTR.
(2) The fall will be considered as a “possible faint” if one of the following conditions is satisfied:
  (a) SBP falls more than 20 mmHg in comparison to SBP 60 s before
  (b) DBP falls more than 10 mmHg in comparison to DBP 60 s before
  (c) HR falls more than 10 in comparison to HR 60 s before
(2) If a “possible faint” is detected a CAP alert notifying this event is sent to the corresponding agent. Otherwise, a CAP message will be sent notifying the previously detected fall event.

## 6. Evaluation of the Proposed System

This Section is focused on providing an evaluation of critical aspects of the system described in previous Sections in order to validate the concepts theoretically described. The evaluation tests address mainly aspects related to the working issues of the core of this system, *i.e.*, a Smart Gateway running an instantiation of *µ*WoTOP. According to the obtained evaluation results, some technical tips are provided with respect to deployment and implementation features behind the real-world scenarios based on *µ*WoTOP.

### 6.1. Test Bench Specification

We have designed a test bench to evaluate *µ*WoTOP global performance. The test bench consists of a simulation environment which allows testing those parameters that are most likely to affect the performance and reliability of the whole system in a real scenario.

**Figure 11:**
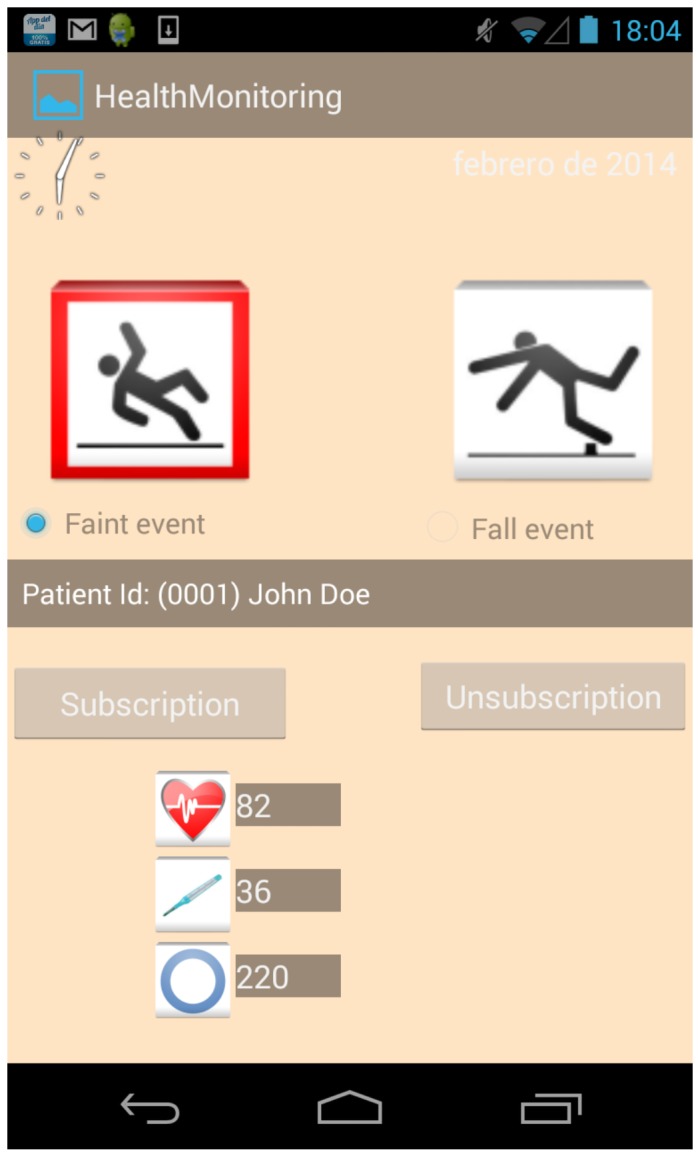
Snapshot of the management application for the medical staff running on Android 4.3.

The scenario to be simulated encompasses specific features that facilitate the evaluation of the whole system through different hypothetical situations with incremental demanding levels. Specifically, the scenario to be simulated consists of a medium size residential care place, which hosts elder people; a restricted number of those people are considered outpatients of a clinic center due to the fact that they suffer from some diseases that does not impede them to develop an independent living by using a system based on *µ*WoTOP. Thus, outpatients wear a BAN with the characteristics commented in the Section 5. A smartphone managed by a carer of the residential care place works as a Smart Gateway running an instantiation of the *µ*WoTOP. Let us suppose all the outpatients wearing the corresponding BAN are under the influence of the Bluetooth signal of the carer’s smartphone (about 30 m). The parameters characterizing the services of this scenario that are able to compromise the system (specially, generated events and subscriptions to those events) are progressively growing until overloading the Smart Gateway in order to obtain the maximum performance and reliability levels of the system. The entities used to simulate such scenario are the following ones:

- *A Smartphone running µWoTOP (Smart Gateway)*: We used a smartphone Galaxy Nexus (ARM Cortex-A9 core duo 1.2 GHz and 16 Gb) connected to a private Wireless Local Area Network (WLAN) at 100 Mbps. A *µ*WoTOP instantiation is running in a background process. The operating system of this smartphone is Android 4.3.
- *Event generators*: Event generators are software components that simulate a set of BANs worn by outpatients which generate events at a given rate (“incoming events”). In our experiments every generator produced 0.5 event/s that were sent to the Smart Gateway . In this way, the simulated environment was much more demanding than a typical real environment, *i.e.*, less than 3 fall/faint per day.
- *Event consumers*: Event consumers are software components simulating agents that subscribe to events dispatched by the generators (“outgoing events”). In our tests, they physically run on different user devices (smartphones and PCs) connected to the same WLAN, and they are subscribed to the events generated by one or more BANs depending on the type of agent to be simulated by the event generator: health monitoring, notification or ambient. Figure 11 shows a snapshot of an application running the health monitoring agent.

These entities were strategically deployed in order to simulate the scenario with different demand needs. The purpose of modeling those simulated environments was to set up several batteries of tests to evaluate the system. Specifically, those batteries of tests were focused on evaluating the influence of three parameters on the scalability, performance and reliability of a *µ*WoTOP-based system: *(i)* the incoming event rate reaching the Smart Gateway; *(ii)* the outgoing event rate that is generated by the Smart Gateway to dispatch events to the consumer agents subscribed to them; and *(iii)* the number of subscriptions managed by the *Event Management Subsystem*.

**Figure 12:**
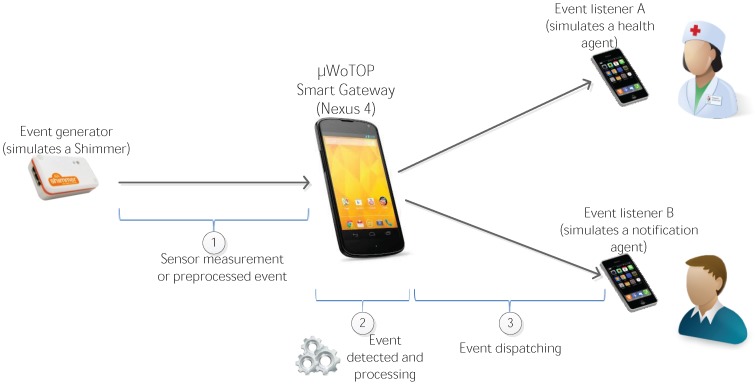
Segments defined to calculate partial delays of the events from the source to the destination.

Basically, the parameters that were measured during the tests were *(i)* the delay when dealing with events and *(ii)* the rate of successfully delivered events to consumer agents from those sent by the Smart Gateway. Figure 12 shows the possible segments of the infrastructure that concentrate the most part of the delay in the whole system.

The segment *1* consists of a direct link established to send few bytes of data from sensors. Theoretically, the delay in that segment is several orders of magnitude less than the delay produced in segments *2* and *3* of the path followed up by the event message shown in the Figure 12.

### 6.2. Evaluation Results and Conclusions

The evaluation tests consisted of measuring the influence of different configurations of the incoming event rate as well as the subscription table managed by the Smart Gateway. The two batteries of test carried out in this evaluation are explained below.

#### (*A*) **Fixed incoming event rate → incremental subscription table** 

The first battery of tests consisted of measuring the influence of the size of the subscription table managed by the *Event Management Subsystem* while the incoming event rate (from the producers to the Smart Gateway) was keeping constant. This means that we are going to vary the number of consumer applications (e.g., carer apps or smart objects) subscribed to a fixed number of event generators (outpatients), also modifying the outgoing event rate. Let us suppose there are only 8 outpatients generating events as specified before (0.5 event/s). Thus, the total rate of events generated by the set of outpatients wearing a BAN in this simulated scenario is 4 event/s. Regarding to the consumer entities subscribed to those events, let us consider a variable list of subscriptions as follow (all subscriptions listed below will be done for every single outpatient involved in these tests):

- 1 subscription from a *health monitoring agent* deployed on a simulated smartphone of a single doctor.
- 5 subscriptions from *ambient agents* to be deployed on smart things.
- 2*n* of subscriptions sent from notification agents deployed on simulated smartphones of outpatient’s relatives, with *n* an integer ranging between 1 and 10.

The minimum number of subscriptions per outpatient (8 subscriptions) was introduced in order to eliminate those scenarios in which few subscriptions are involved, since they do not provide much information for the evaluation of the system (Between 1 and 7 subscriptions, outgoing event rate to be generated does not jeopardize the Smart Gateway with respect to both performance and reliability, so obtained results are pretty similar between them.). Thus, the minimum number of subscriptions in the subscription table was 64 and the maximum number of subscriptions 208.

According to the subscription lists described above and keeping in mind that the incoming event rate was set up to 4 event/s for this battery of tests, the total rate of events delivered to all subscribed agents was (6 + 2*n*)4 events/s, and thus ranged from 32 to 104 event/s in steps of 16 event/s. In this battery of tests, 10 tests were performed in total, each one for a different value of *n*. During each test, 6,000 events (“incoming events”), at a rate of 4 events/s, were generated and gathered by the Smart Gateway. Those events, in turn, generated "outgoing event" rates to be dispatched to the corresponding consumer agents according to the subscription table that was set up in each test. For every single test, the delay generated by dispatching each of the 6,000 events (It was measured from the moment an event reaches the Smart Gateway until it is delivered to the last of the consumer agent that were subscribed to it.) was registered in order to calculate their average value and typical deviation.

**Figure 13:**
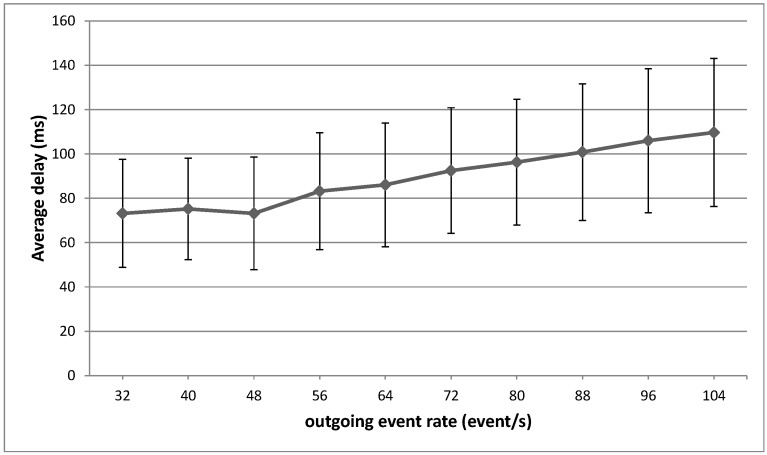
Average value and standard deviation of the delay registered for each of the 10 tests in the first battery of tests.

Figure 13 shows the average delay of the messages (corresponding to the segments 2 and 3 indicated in Figure 12) as a function of outgoing event rate. The minimum average delay (73 ms) was registered when the Smart Gateway generated 32 event/s. On the other hand, the maximum delay (109 ms) was registered when the Smart Gateway generated 104 event/s. As expected, the delay for delivering events increases for an increasing number of subscriptions in the subscription table. In all cases the observed delay was acceptable even for a type of applications with hard *real-time* requirements as the one proposed in this case study (an in Section 3).

**Figure 14:**
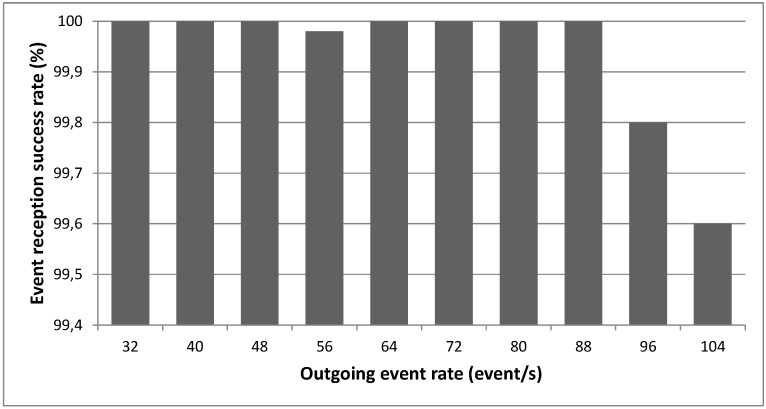
Event reception success rate registered for different outgoing event rates.

As commented before, we also registered the success rate of event effectively delivered to the consumer agents which subscribed to the events. This is a significant factor that indicates the reliability level of the system and, specifically, the reliability of the Smart Gateway when dealing with different sizes of the subscription table. The obtained results with respect to the event reception success rate are shown in Figure 14.

These results reflect reliability levels that reach 100% in most of the cases. In the worst case, the reliability level is lightly under values of three 9’s which is still a good reliability level even for an ehealth application. To demonstrate this claim, let us take the worst case, the test generating 104 events/s (corresponding to 208 subscriptions, *i.e.*, 26 subscription per outpatient). Taking into account that a total of 6000 events were generated, 156,000 events had to be theoretically delivered to the consumer agents, but 155,376 events actually reached its destination. One can think that in a real scenario in which, for example, 2 events per minute are generated, to lose 1 event every 125 min could be a bad reliability level. Nothing further from reality, since such a real scenario is much lower demanding than the environment simulated in this test, with respect to outgoing event rate. Thus the expected reliability would be much higher in that real scenario since any bottleneck will appear.

Regarding the scalability level of the system, we claim that the Smart Gateway deployed on a Nexus 4 was very satisfactory until the maximum number of subscriptions used in this battery of tests (208). The difference between the delays obtained in the first test and the last test is lower than 30 ms. Thus, we can expect that the proposed system would scale quite well in a medium size residential care place, as the one characterized in this simulated environment. Let us suppose that, in a real scenario, 8 subscriptions per outpatients are enough to provide a good assistance level, then the proposed system could assume until 24 outpatients, 3 times more than the simulated in this battery of tests.

#### (*B*) **Fixed subscription table → incremental incoming event rate** 

The second battery of tests consisted of evaluating the behaviour of the system in terms of event delay and event reception success rate, as before, but, this time, keeping constant the size of the subscription table while increasing the incoming event rate. We carried out five test series in which a fixed size of the subscription table was set up. Specifically, those test series were carried out using from 6 to 10 subscriptions, in steps of 1 subscription, so 5 test series were performed in total. The variable incoming event rate were set up by simulating 8 outpatients generating events at different rates, as shown in Table 4.

**Table 4:** Incoming event rate generated for each of the five test series in the second battery of tests.

Test number	Event rate per outpatient(event/s)	Total incoming event rate (event/s)
1	0.25	2
2	0.5	4
3	0.75	6
4	1	8
5	1.25	10
6	1.5	12
7	1.75	14

**Figure 15:**
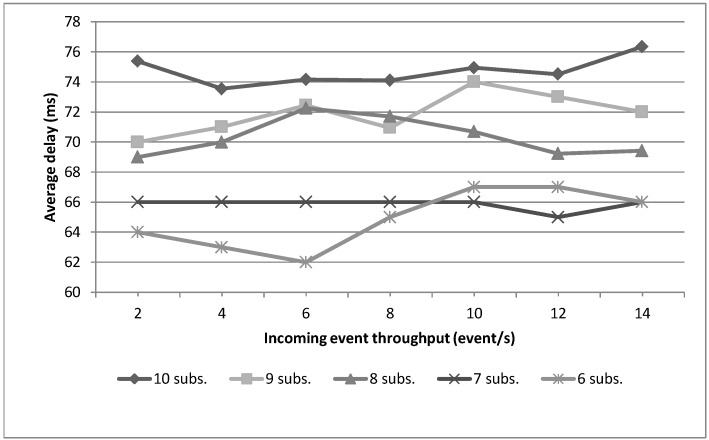
Average delay for every tests case in the second battery of tests.

Similarly to the first battery of tests, during every test case 6,000 events were generated in total and sent to the Smart Gateway in different experiments as indicated in Table 4. In turn, those events were dispatched at different outgoing event rates, depending on the number of consumer agents subscribed to them. In these experiments, we also registered the delay produced by dispatching all the 6,000 events in order to calculate their average value. Results are shown in Figure 15.

Analysing these results, we can conclude that delays of delivered events are quite stable within the same set of test series, varying no more than 5 ms. As expected, the average delay increases gradually as the subscription table grows. Clearly, the size of the subscription table has more impact on event delay than the incoming event rate that is transmitted to the Smart Gateway. This characteristic makes the Smart Gateway potentially resistant under situations in which the incoming event rate shoots up quickly and unexpectedly, e.g., an emergency involving several outpatients.

**Figure 16:**
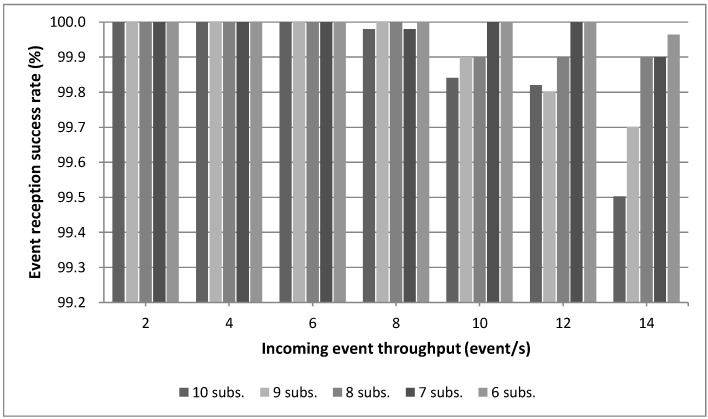
Event reception success rate registered for each of the test cases.

Regarding the reliability level observed in this battery of tests, the results are similar to the ones obtained in the first battery of tests. The results related to the success rate of event correctly delivered to consumer agents are represented in Figure 16.

In conclusion, the reliability level seems to be directly related to the outgoing event rate. On the other hand, the size of the subscription table does not seem to have too much impact in the reliability of the system. This fact can be clearly observed in those tests cases in which incoming event rates are low (between 2 and 6); in those test cases the outgoing event rate is also relatively low, and the success rate of delivered events is 100%, without exception.

## 7. Conclusion and Further Work

In this paper a novel WoT-based approach has been proposed aimed at contributing to the state of the art of techniques, mechanisms and tools that try to achieve successful deployments of modern and future smart spaces. Firstly, we explored the related work in order to analyse the challenges and opportunities that are motivating current research and approaches in the field of the IoT and WoT, specifically those focused on the deployment of heterogeneous ecosystems of embedded devices. From the analysis of related work, we have outlined some ehealth-based scenarios involving smart spaces that are currently available or will be available in the near future with the aim at analysing their major functional and non-functional requirements. Our analysis was focused on a wide range of applications designed to provide support in a variety of situations: from those dedicated to overcome serious health situations of outpatients (emergencies or diseases supervision) to those used just to advice people on how to achieve a healthier lifestyle.

According to the requirements collected in the mentioned analysis, we established the pillars of the main contribution of this paper: a WoT-based platform to facilitate the deployment of enriched and heterogeneous smart spaces, so called *micro Web of Thing Open Platform* (*µ*WoTOP). The *µ*WoTOP is a lightweight open platform that aims at enabling mobile and resource-constrained devices to work as smart gateways of smart spaces. These smart spaces could be composed of heterogeneous ecosystems of embedded sensor and actuator devices deployed on the user’s environment. The *µ*WoTOP architecture is based on an effective combination of the WoT and the IoT paradigms. This particular architectural feature improves significantly the pervasiveness of the whole system, including the mobility in smart spaces as well as the connectivity between embedded devices and Web-based services.

In order to proof the theoretical approach presented in this paper, we implemented a ubiquitous ehealth system to provide outpatients with specialized health assistance at their own homes or residential care places. The infrastructure of this system was essentially composed of Body Area Networks, a *µ*WoTOP-based Smart Gateway and several agents that consumed context information by means of event-driven mechanisms. To this purpose, we set up a simulated environment that performed an exhaustive test-bench of the whole system; two battery of tests were performed in order to collect measurements related to the system performance (latency and reliability) when dispatching events from a Smart Gateway to consumer agents. Performance results proved that a *µ*WoTOP-based system could be suitable to work in a medium-scaled real scenario even in those with hard or firm real-time requirements. For example, we conclude that a feasible scenario could consist of 24 outpatients with 8 agents consuming events from each one of them, and a smartphone Nexus 4 working as a Smart Gateway, in case the outgoing event rate does not exceed 4 event/s. In general, the scalability of a *µ*WoTOP-based system depends on the incoming and outgoing event rate that must manage the Smart Gateway. Nevertheless, more than one Smart Gateway can be deployed within a smart space in order to increase its scalability in terms of number of supported outpatients and consumer agents, as well as in terms of incoming and outgoing event rates.

For future work, we are reviewing and improving some architectural features of the *µ*WoTOP in order to optimize the performance, specially the reliability of the *µ*WoTOP-based Smart Gateways when dealing with high demanding situations (high incoming and outgoing event rates, large subscription tables, *etc.*). The idea is to include a mechanism to automatically retransmit data when they are lost or an inconsistency is detected. Additionally, we will perform more evaluations in which more than one Smart Gateway are involved in order to design mechanisms to balance automatically the workload. The objective will be to guarantee good Quality of Service levels automatically according to the scalability requirements of the smart space at different points in its life-cycle.
